# The effect of age on ankle versus hip proprioceptive contribution in balance recovery: application of vibratory stimulation for altering proprioceptive performance

**DOI:** 10.1007/s13534-024-00451-7

**Published:** 2025-01-03

**Authors:** Mehran Asghari, Karam Elali, Nima Toosizadeh

**Affiliations:** 1 Department of Biomedical Engineering, University of Arizona, Arizona, USA; 2 Department of Rehabilitation and Movement Sciences, School of Health Professions, Rutgers Health, Rutgers University, Newark, NJ 07107 USA; 3 Department of Neurology, Rutgers Health, Rutgers University, Newark, NJ USA; 4 Brain Health Institute, Rutgers University, New Brunswick, NJ USA

## Abstract

While tripping is the leading cause of injurious falls in older adults, the influence of ankle and hip proprioceptive information in balance recovery among older adults is still not clearly understood. The objective of this study was to assess the influence of ankle vs. hip proprioceptive information by altering muscle spindle performance using vibratory stimulation among older adults and healthy young control participants. Two groups of young (*n* =  20, age =  22.2 ± 3.1 years) and older adult (*n*  =  33, age = 74.0 ± 3.8 years) participants were recruited and went through treadmill perturbation (sudden backward treadmill movement mimicking a trip), while they were equipped with vibratory devices (no vibration, and 40 and 80 Hz) on either ankle or hip muscles. Kinematics of the recovery were measures using motion sensors on lower extremities and the trunk. Results showed that vibratory stimulation on ankle significantly influenced balance recovery response (i.e., increased reaction time by 18% and increased recovery step length by 21%) among healthy young control, while it showed no effect when placed on hip muscles. On the other hand, while vibratory stimulation on ankle showed no effect on balance recovery among older adults, it significantly influenced balance recovery when applied to the hip muscles (i.e., increased reaction time by 12% and increased recovery step length by 10%). Current findings suggest that the role of ankle vs. hip proprioceptive information in balance recovery may change by aging. Findings may potentially be used for targeting the appropriate location for balance interventions and reducing the fall risk in older adults.

## Introduction

Falls are the primary cause of traumatic injury in older adults, with tripping being the leading cause. Human balance relies on sensory units, muscle reflexes, and the central nervous system and alterations in these components due to aging or diseases can lead to balance deficits [1, 2]. Several studies have shown that deficits in postural control and muscle strength are significant intrinsic risk factors for falls. Adequate training regimens designed to counter these factors have the potential to reduce the rate of falling in older adults [3–5]. While various methods have been established to assess and improve balance recovery by targeting lower-extremity muscle strength deficits [6], little is known about the distinct roles of ankle and hip proprioception during dynamic balance recovery in older adults, since previous studies have explored proprioceptive control during upright standing balance [7]. Indirect evidence suggests that proprioception deficits are highly associated with poor balance recovery from tripping [8–11]; however, a robust method for understanding aging-related proprioceptive deficits is lacking.

To maintain balance, muscle proprioceptive inputs are required to provide the central nervous system with information regarding the position of each segment [12]. Two factors associated with balance recovery deterioration among older adults are delayed placement of the recovery leg and delayed moment generation in the stance leg [8]. In both factors, proprioception from the ankle and hip joint muscles plays a critical role in feedback (reactive) and feedforward (preparatory) control, and the regulation of muscle stiffness to achieve specific movement acuity, coordination, and balance, resulting in a quick and efficient recovery response [10]. Yet, the direct consequences of aging-related deficits in proprioceptive information from hip and ankle muscle groups during balance recovery are not well understood. Proprioceptive information from the ankle and hip is vital for maintaining balance during upright standing, and there are alterations in implications of these proprioceptive sources by age [13, 14].

In addition to upright standing, previous work, based on results from muscle activity onsets, suggested that proprioceptive information from the hip musculature may involve in initiating the recovery response, whereas ankle proprioception information may contribute to establishment of the recovery step [15, 16]. Further, proprioceptive input from these areas plays a critical role in both reactive and preparatory control, with evidence suggesting that ankle proprioception is more engaged in less demanding balance tasks, while hip proprioception becomes crucial during more challenging conditions [17]. Other studies have shown that younger adults often rely on ankle proprioception to maintain balance under mild perturbations [18, 19], whereas older adults tend to shift to a hip-centered strategy as ankle proprioception declines with age [19]. Another study demonstrated that older adults with reduced balance capacity depend more on hip proprioception to compensate for ankle proprioceptive deficits, especially under dynamic conditions that challenge balance [20]. However, there remains limited research differentiating how ankle and hip proprioception specifically contribute to dynamic balance recovery in older adults, particularly in scenarios that mimic real-life fall risks. To describe the distinct contributions of ankle versus hip proprioceptive inputs in dynamic balance recovery, direct methods for assessing proprioceptive performance, rather than muscle activity onsets, would be invaluable. Further, it is important to learn how this contribution would alter with aging.

Previous research has indicated that vibratory stimulation can effectively influence proprioception, particularly muscle spindle function [21–26]. In our earlier studies, we observed differences in the effect of vibratory stimulation among young versus older adults for standing balance and timed-up-and-go (TUG) performance. Additionally, our investigations in healthy young participants elucidated the role of proprioceptive input from ankle and hip muscles in balance recovery, identifying key parameters influenced by vibratory stimulation during dynamic tasks of balance recovery within treadmill perturbation [27]. Among control young adults, similar to its effects on upright standing balance, vibratory stimulation significantly impacted balance recovery, with distinct contributions observed between ankle and hip joints [27]; stimulation on ankle muscles significantly altered balance performance of reaction time and recovery stepping in young participants, while hip stimulation showed no significant effect.

In continuation of previous research, the objective of this study was to assess the effect of vibratory stimulation on ankle versus hip muscles among older adults, in comparison to healthy young control. Our hypothesis posits that vibratory stimulation alters muscle spindle function and consequently balance recovery following treadmill tripping in older adults; however, due to balance adjustment by aging, unlike control young adults, these effects would be significant only when vibratory stimulation is applied to the hip muscles. This study marks the first attempt to separately investigate the contributions of ankle and hip proprioception in balance recovery in older adults.

## Method

### Participants and clinical measures

We recruited two groups of participants, including control young participants and community-dwelling older adults. Inclusion criteria for the young participants were age between 18 and 30 and the ability to understand study instructions, while exclusion criteria included cognitive impairments (Montreal Cognitive Assessment (MoCA) < 20), mobility disorders, and history of dizziness, vertigo, sedating medication, or alcohol consumption within 24 h of testing. For older adults, inclusion criteria were age 65 years or older and the ability to understand study instructions. Exclusion criteria for older adults included disorders causing severe motor and balance deficits (e.g., stroke, Parkinson’s disease, severe arthritis, lower-extremity amputation, spinal cord pathologies, and diabetes), history of severe vestibular disorders, central nervous diseases, cognitive impairment (MoCA score < 20), vision problems affecting balance, and history of dizziness, vertigo, sedating medication, or alcohol consumption within 24 h of testing. All participants provided written informed consent in accordance with the Declaration of Helsinki, with approval from the University of Arizona’s Review Boards [28].

In addition to demographic information, several questionnaires were collected from older adult participants to account for confounding variables that can potentially influence balance. Questionnaires included Stopping Elderly Accidents, Deaths & Injuries (STEADI) [29, 30], fear of falling using Fall Efficacy Scale-International (FES-I) [31], cognition using MoCA [32], Charlson comorbidity score [33], depression using Patient Health Questionnaire (PHQ-9) with higher scores indicating greater depressive symptoms [34], pain in lower-extremity based on Visual Analog Scale (VAS) [35], and vestibular deficits using Dizziness Handicap Inventory (DHI). DHI evaluates dizziness-related impairment and vestibular symptoms, which are relevant for balance.

### Study design

The demographic information, along with shin and thigh lengths, were measured during data collection to extract balance recovery outcomes. Participants were asked to wear lightweight sport shoes, to provide consistency in footwear and minimize associated confounding effects. There were two types of balance recovery sessions involving vibratory stimulation on either the ankle or hip muscles. To minimize potential learning effects from repeated balance recovery attempts, each participant was randomly assigned to only one of the balance recovery sessions (ankle or hip stimulation). In each session, after two practice trials with slow treadmill perturbation exposures, each participant completed 15 trials of treadmill perturbation within three bouts (Table 1). Participants were exposed to either low or high-frequency vibration or no vibratory stimulation in each bout. There were ~ 5-minute rest periods between trials to minimize potential residual effects of vibration and confounding influence of fatigue. Each bout included four trials of sudden backward belt movement with two difficulty levels, including two trials at a maximum speed of 0.35 m/sec and two trials at a maximum speed of 0.7 m/sec. To minimize anticipation effects, one forward belt movement trial with a maximum speed of 0.2 m/sec, was randomly assigned to each bout (Table 1). The order of bouts (no-vibration, low, and high frequency) and balance recovery difficulty (treadmill speeds) were randomized to minimize potential fatigue, learning, and vibration residual effects.

**Table 1:** Treadmill perturbation exposure

Bout 1: No vibration				
Backward 0.35 m/s	Backward 0.7 m/s	Forward 0.2 m/s	Backward 0.35 m/s	Backward 0.7 m/s

Bout 2: Ankle (or Hip) low frequency vibration				
Backward 0.7 m/s	Forward 0.2 m/s	Backward 0.35 m/s	Backward 0.7 m/s	Backward 0.35 m/s

Bout 3: Ankle (or Hip) high frequency vibration				
Forward 0.2 m/s	Backward 0.35 m/s	Backward 0.7 m/s	Backward 0.35 m/s	Backward 0.7 m/s

Participants were randomly assigned to ankle or hip vibration trials; each participant was only exposed to vibration at one location. The order of bouts (no vibration and low and high frequency vibration) and trials (0.35, 0.7, or 0.2 m/s speed) were randomized

### Vibration area and frequency

Vibratory stimulation was applied bilaterally to ankle area muscles including tibialis anterior, peroneus longus, soleus, and gastrocnemius, or hip area muscles including quadriceps, gluteus medius, and paraspinals. Vibratory stimulation devices were placed on the belly of the muscles, based on SENIAM guidelines for both the ankle and hip joint muscles [36]. These muscles were chosen for ankle and hip joints because proprioceptive information from these muscles is crucial for ankle- and hip-strategy balance mechanisms during static and dynamic balance, and they have also been the targeted muscles for vibratory stimulation in previous research [15, 21–23, 25, 37].

We considered two vibration frequencies for exciting muscle spindles. Previous evidence suggests that in healthy participants, 80 Hz vibrations of ankle muscles produce the maximal effect on postural balance, while frequencies below 40 Hz may not produce consistent effects and can vary between participants [38–40]. Accordingly, we used Gaussian noise, band-limited to 80 Hz for higher and 40 Hz for lower frequency stimulations. Magnetic actuator systems (C-2HDLF Tactor, Engineering Acoustics, FL, USA) and a Universal Controller (TDK, Engineering Acoustics, FL, USA) were used to provide the appropriate frequency ranges. The vibration amplitude was set to 1 ± 0.002 mm, a level found to effectively influence muscle spindle afferents [41, 42].

### Treadmill perturbation setup

**Fig. 1:**
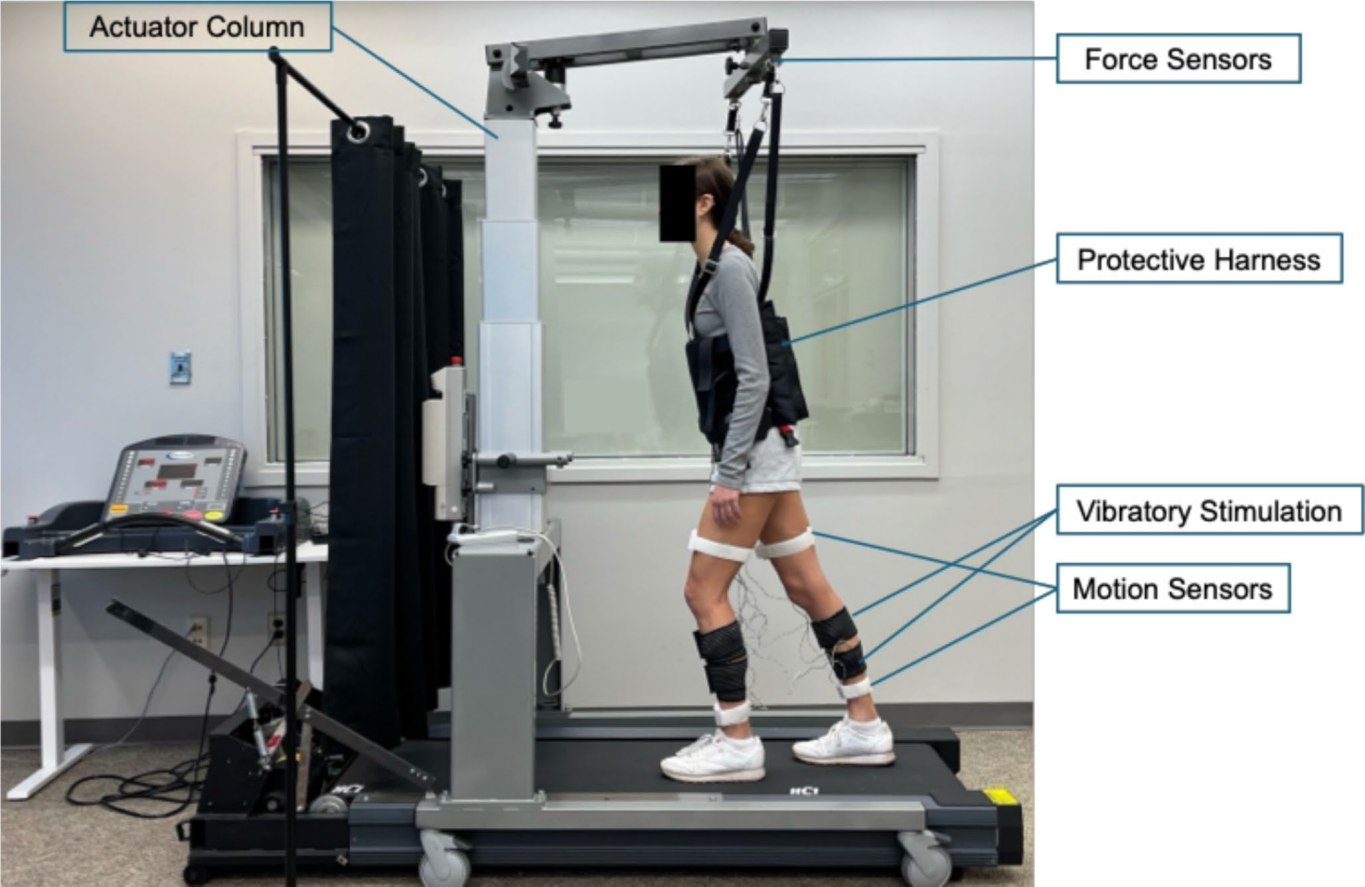
Treadmill perturbation setup during recovery stepping with vibratory stimulation applied to the ankle joint muscles

A modified treadmill setup (PhysioGait & PhysioMill, HealthCare International, Langley, WA, USA) was used to impose trip-like perturbations [10, 43]. To prevent an actual fall, the PhysioGait provides a protection harness that prevents knee or hand contact with the treadmill (Fig. 1). The harness was equipped with force transducers to measure the amount of weight tolerated with the harness. Participants were asked to stand motionless on the treadmill, which would start running unexpectedly. The perturbation involved a sudden backward movement of the belt to move the feet posteriorly and induce a forward loss of balance, similar to tripping [10, 44]. In response, the sensorimotor system executes a reactive stepping to expand the base of support and establish stable walking afterwards. Participants wore the harness, adjusted to prevent falling but not interfere with movement, and were instructed not to use their hands for support, as this would invalidate the trial. In each trial, the treadmill reached the max speed in ~ 37 ± 5 msec. Treadmill max speeds and acceleration were selected based on previous studies [10, 27]. Following a successful recovery, participants walked until they regained their steady-state walking, which included a minimum of 20 steps.

### Balance recovery outcomes

Failing to recover from the perturbation was identified when the entire body weight was supported by the harness [10, 43]. Recoveries with integrated weight support greater than 5% of the body weight × second, from toe off to heel strike of the recovery stepping, were classified as harness-assisted recoveries [43]. All other recoveries were considered successful and were used for calculating balance recovery outcomes. Three-dimensional acceleration and angular velocity of shins, thighs, and the trunk were measured using five wearable motions sensors (LEGSys™, BioSensics LLC, Boston, MA, USA, sampling frequency = 100 Hz), to derive balance recovery and gait outcomes [45, 46]. The signals from the sensors were filtered using first-order high pass butter-worth filter with a cutoff of 2.5 Hz, to remove noise and drift. Balance recovery outcomes included response time, recovery step length, trunk angle during toe-off and heel-strike of recovery stepping, and the required time for full recovery (Table 2).

**Table 2:** Balance recovery outcomes

Outcome	Definition
Reaction time	Time from the onset of treadmill motion to recovery step toe-off
Recovery step length	Length of initial step as the percentage of the body height
Toe-off trunk angle	Trunk angle in the sagittal plane at the onset of recovery step toe-off
Contact trunk angle	Trunk angle in the sagittal plane at the recovery step ground contact
Full recovery time	Time to reach steady-state walking as defined by the first stride of the group of six strides with an SD below the median SD

SD, standard deviation

### Statistical analysis

Within each group of young and older adults, demographic information and clinical measures were compared between participants with ankle and hip exposures using analysis of variance (ANOVA) models for continuous variable and using Chi-square analysis for gender distributions. The association between balance recovery outcomes (Table 2) and vibration conditions (no vibration, 40 Hz, and 80 Hz vibration) was determined using repeated measures mixed effects models. Within these models, independent variables of vibration frequency and treadmill speed, along with their interaction effect, were considered as within-subject factors; sex was considered as a between-subject variable. Effect sizes were calculated using Cohen f or d calculations, using G*Power (version 3.1.9.7, copyright 2020, University of Dusseldorf, Germany). This analytical approach was applied separately for ankle and hip stimulations. Our primary focus was on main and interactive effects involving vibration conditions, with the main effects of speed on outcome measures not explicitly reported. All analyses were done using JMP (Version 14, SAS Institute Inc., Cary, NC, USA), and statistical significance was concluded when *p* < 0.05 based on the sample size and exploratory nature of the current study.

## Results

### Participants

Twenty control young participants were recruited. Ten participants completed the ankle stimulation (five males and five females, age = 21.5 ± 3.0 years) and 10 participants completed the hip stimulation (four males and six females, age = 22.8 ± 2.5 years). On the other hand, 33 older adult participants were recruited, among whom, 16 completed the ankle stimulation (7 males and 9 females, age = 73.9 ± 3.7) and 17 completed the hip stimulation (6 males and 11 females, age = 74.1 ± 4.0). All trials for both groups were deemed successful recoveries. None of the demographic and clinical measures were significantly different between two groups with ankle vs. hip vibration exposures for both young and older adult participants (*p* > 0.11, Table 3). Three older adult participants within each of ankle and hip exposure group scored between 23 and 25 on the MoCA test, classifying them as mild cognitively impaired; the rest were cognitively healthy. All older adult participants within both ankle and hip exposure groups were classified as low fall risk. None of the participants had history of dizziness, severe lower extremity pain, or severe comorbidities that would exclude them from the trials (Table 3). Of note, the data of healthy young participants were published before by the same group and are presented here for comparison with older adult participants [27].

**Table 3:** Demographic information and clinical measures data and comparisons between two groups with ankle vs. hip exposures

Demographics	Control young (*n* = 20)			Older adults (*n* = 33)		
	Ankle	Hip	*p*-value (effect size)	Ankle	Hip	*p*-value (effect size)
Number, n (% of the total)	10 (50%)	10 (50%)	-	16 (48%)	17 (52%)	-
Male, n (% of the group)	5 (50%)	4 (40%)	0.65 (0.20)	7 (44%)	6 (35%)	0.62 (0.18)
Age, year (SD)	21.5 (3.0)	22.8 (2.5)	0.34 (0.47)	73.9 (3.7)	74.1 (4.0)	0.86 (0.05)
Weight, kg (SD)	79.29 (12.79)	69.71 (11.23)	0.11 (0.79)	68.62 (18.08)	65.17 (16.69)	0.57 (0.20)
Height, m (SD)	1.71 (22.91)	1.70 (10.35)	0.87 (0.01)	1.69 (0.12)	1.68 (0.12)	0.69 (0.08)
BMI, kg/m^2^ (SD)	28.35 (10.44)	24.09 (3.88)	0.12 (0.54)	23.31 (3.89)	22.64 (3.77)	0.62 (0.17)

Clinical measures				Ankle	Hip	*p*-value
STEADI score, 0–14 (SD)	-	-	-	1.06 (1.24)	1.00 (1.22)	0.89 (0.05)
FES-I, 16–64 (SD)	-	-	-	18.13 (2.13)	18.06 (2.08)	0.93 (0.03)
MoCA, 0–30 (SD)	-	-	-	27.50 (2.00)	27.50 (2.00)	0.99 (0.00)
Comorbidity, 0–37 (SD)	-	-	-	1.56 (1.50)	1.47 (1.50)	0.86 (0.06)
PHQ-9 0–27 (SD)	-	-	-	0.94 (1.91)	0.88 (1.87)	0.93 (0.03)
Pain, 0–10 (SD)	-	-	-	1.00 (2.28)	1.18 (2.32)	0.83 (0.08)
DHI score, 0-100 (SD)	-	-	-	1.38 (2.60)	1.29 (2.54)	0.93 (0.04)

STEADI, stopping elderly accidents, deaths & injuries; FES-I, fall efficacy scale-international; MoCA, montreal cognitive assessment; PHQ-9, patient health questionnaire; DHI, dizziness handicap inventory

### Ankle joint vibratory stimulation

Among the control young group, ankle joint vibratory stimulation significantly affected reaction time (increased by 18%) and recovery step length (increased by 21%) across different conditions (*p* < 0.002, Table 3). Other balance recovery outcomes, including toe-off trunk angle, contact trunk angle, and full recovery time (defined in Table 2), showed no significant change due to ankle vibratory stimulation in control young participants (*p* > 0.07, Table 4). For older adult participants, ankle vibratory stimulation showed no significant effect on any of the balance recovery outcomes (*p* > 0.68). In both young and older adults, significant influences of treadmill speed were observed on all balance recovery outcomes (*p* < 0.05), except the full recovery time for both young and older adult groups (Table 4). There were no significant interaction effects between vibration frequency and treadmill speed on any balance recovery outcomes (*p* > 0.12).

**Table 4:** Differences in balance recovery outcomes across three conditions of no-vibration, 40 hz vibration, and 80 hz vibration for the ankle joint for healthy young and older adult participants

Outcome	Slow speed (0.35 m/s)			Fast speed (0.7 m/s)			Speed	Frequency
	No stimulation	40 Hz Stimulation	80 Hz Stimulation	No stimulation	40 Hz Stimulation	80 Hz Stimulation	*p*-value	*p*-value
*Ankle joint vibratory stimulation for control young*								
Reaction time (sec)	0.344 (0.132)	0.418 (0.164)	0.416 (0.146)	0.258 (0.021)	0.266 (0.032)	0.322 (0.073)	< 0.0001* (1.00)	0.0014* (0.34)
Recovery step length (cm)	12.027 (4.872)	14.735 (5.807)	15.255 (4.463)	17.066 (4.729)	19.515 (4.908)	20.644 (5.715)	< 0.0001* (0.99)	< 0.0001* (0.43)
Toe-off trunk angle (deg)	3.867 (2.391)	3.526 (3.122)	4.440 (3.317)	4.716 (2.759)	4.374 (2.095)	5.127 (3.855)	0.0465* (0.28)	0.2266 (0.20)
Contact trunk angle (deg)	3.947 (2.964)	4.165 (3.261)	5.037 (4.386)	7.222 (4.535)	6.986 (4.032)	7.406 (4.406)	< 0.0001* (0.72)	0.3796 (0.20)
Full recovery time (sec)	1.463 (0.738)	1.179 (0.769)	1.962 (1.438)	1.539 (1.085)	1.611 (1.123)	1.822 (1.036)	0.6272 (0.21)	0.0739 (0.50)
*Ankle joint vibratory stimulation for older adults*								
Reaction time (sec)	0.308 (0.063)	0.299 (0.068)	0.316 (0.072)	0.291 (0.043)	0.299 (0.060)	0.293 (0.058)	0.0335* (0.22)	0.6777 (0.17)
Recovery step length (cm)	11.054 (3.532)	13.411 (5.047)	12.014 (3.591)	16.813 (4.221)	14.642 (3.927)	15.537 (3.785)	< 0.0001* (0.90)	0.7888 (0.38)
Toe-off trunk angle (deg)	5.880 (2.365)	6.500 (3.782)	5.900 (2.672)	8.246 (3.796)	7.484 (3.500)	8.093 (3.708)	< 0.0001* (0.57)	0.9300 (0.13)
Contact trunk angle (deg)	7.312 (4.358)	8.711 (6.480)	7.025 (4.565)	13.408 (5.558)	11.767 (6.442)	13.531 (5.602)	< 0.0001* (0.99)	0.9381 (0.21)
Full recovery time (sec)	1.553 (1.177)	1.878 (1.236)	2.207 (2.715)	1.748 (1.012)	1.693 (1.029)	1.773 (0.993)	0.4386 (0.18)	0.2971 (0.25)

SD, standard deviation

### Hip joint vibratory stimulation

**Fig. 2:**
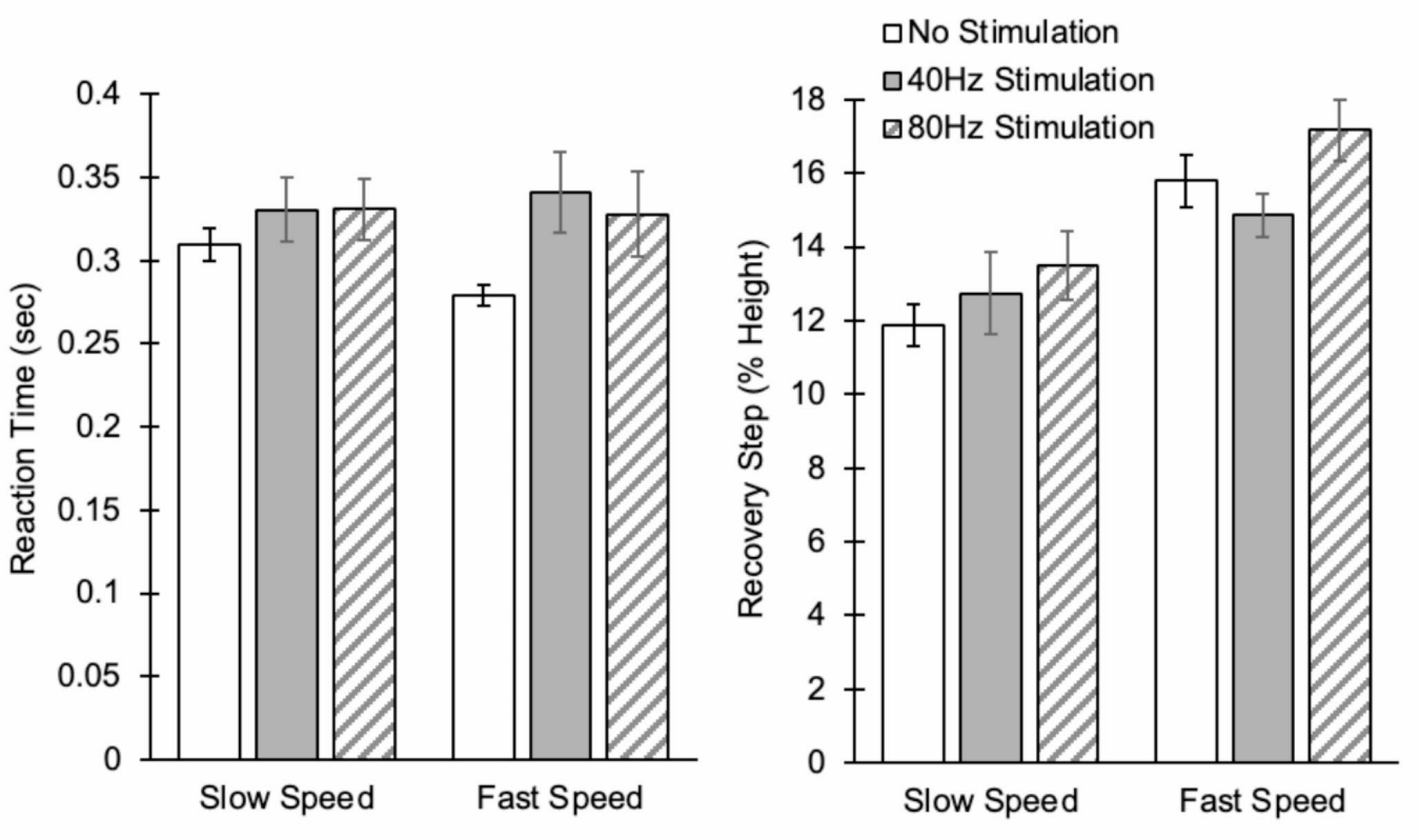
Changes in reaction time and initial recovery step length using vibratory stimulation on the hip joint for older adults

Among control young participants, hip joint vibratory stimulation, among all balance recovery outcomes, only significantly altered the full recovery time (Table 5). On the other hand, among older adult participants, hip vibratory stimulation significantly increased the reaction time by 12% across the conditions (Table 5; Fig. 2). Recovery step length also significantly increased when older adults were exposed to vibratory stimulation (average 10% increase across conditions, Table 5; Fig. 2). None of the other balance recovery outcomes was significantly different across vibration conditions for older adults (*p* > 0.39, Table 5). Similar to the ankle joint results, in both groups, significant influences of treadmill speed were observed on all balance recovery outcomes, except the full recovery time for both young and older adult groups, as well as reaction time in older adults (*p* > 0.23, Table 5). No significant interaction effect between vibration frequency and treadmill speed was observed for any balance recovery outcomes (*p* > 0.07).

**Table 5:** Differences in balance recovery outcomes across three conditions of no-vibration, 40 hz vibration, and 80 hz vibration for the hip joint for healthy young and older adult participants

	Slow speed (0.35 m/s)			Fast Speed (0.7 m/s)			Speed	Frequency
Outcome	No stimulation	40 Hz stimulation	80 Hz stimulation	No stimulation	40 Hz stimulation	80 Hz stimulation	*p*-value (effect size)	*p*-value (effect size)
*Hip joint vibratory stimulation for control young*								
Reaction time (sec)	0.376 (0.139)	0.392 (0.166)	0.394 (0.163)	0.250 (0.055)	0.265 (0.058)	0.305 (0.133)	< 0.0001* (0.94)	0.3210 (0.08)
Recovery step length (cm)	13.393 (4.205)	14.435 (3.813)	13.645 (3.356)	18.017 (4.237)	17.477 (4.269)	17.143 (4.659)	< 0.0001* (0.90)	0.7204 (0.18)
Toe-off trunk angle (deg)	3.902 (1.789)	4.701 (1.796)	4.142 (1.726)	8.118 (2.079)	7.040 (2.345)	7.265 (2.626)	< 0.0001* (1.57)	0.7952 (0.30)
Contact trunk angle (deg)	3.277 (2.545)	3.815 (2.965)	3.864 (2.888)	7.699 (2.163)	6.873 (3.782)	7.738 (4.074)	< 0.0001* (1.129)	0.6706 (0.14)
Full recovery time (sec)	1.155 (1.105)	1.864 (1.517)	2.306 (1.673)	1.395 (1.068)	1.825 (1.249)	1.802 (1.175)	0.6602 (0.20)	0.0188* (0.54)
*Hip joint vibratory stimulation for older adults*								
Reaction time (sec)	0.310 (0.060)	0.331 (0.112)	0.331 (0.107)	0.279 (0.037)	0.341 (0.141)	0.328 (0.148)	0.6764 (0.24)	0.0290* (0.16)
Recovery step length (cm)	11.877 (3.330)	12.746 (6.478)	13.509 (5.411)	15.804 (4.041)	14.866 (3.488)	17.174 (4.860)	< 0.0001* (0.73)	0.0239* (0.22)
Toe-off trunk angle (deg)	6.075 (2.668)	6.548 (2.354)	5.803 (2.605)	7.762 (2.571)	8.083 (2.635)	7.868 (2.247)	< 0.0001* (0.70)	0.3899 (0.20)
Contact trunk angle (deg)	6.602 (3.562)	7.982 (5.259)	6.069 (4.231)	11.197 (4.231)	11.036 (3.542)	12.175 (4.541)	< 0.0001* (1.08)	0.6437 (0.28)
Full recovery time (sec)	1.763 (1.759)	2.561 (3.654)	2.686 (3.977)	2.016 (1.008)	1.826 (0.926)	1.940 (0.841)	0.2301 (0.23)	0.5446 (0.20)

SD, standard deviation

## Discussion

### Vibratory stimulation effects

In the current study, we explored the role of proprioceptive information from the ankle and hip muscles in balance recovery performance, in young and older adults. As hypothesized the vibratory stimulation significantly influenced the balance recovery performance in older adults (based on changes in the reaction time and recovery step length as the two major recovery parameters). We further confirmed that the location of stimulation plays an important role on its effects on balance recovery performance. In our previous research we indicated that vibratory stimulation on ankle significantly influence immediate balance recovery response (i.e., reaction time and recovery step length) among healthy young control, while it showed no effect when placed on hip muscles [27]. On the other hand, while vibratory stimulation on ankle showed no effect on balance recovery among older adults, stimulation significantly influenced balance recovery when hip muscles were exposed to it. This finding, for the first time, confirmed that vibratory stimulation influences balance recovery by altering proprioceptive performance, and the level of the effect among young vs. older adults depends on the location of the stimulation exposure.

Building upon previous studies demonstrating significant alterations in upright postural balance due to vibratory stimulation, the current study showed that stimulation of ankle and/or hip muscles can alter balance recovery performance as well. The main theory is that vibratory (or electrical) stimulation influences proprioceptive performance by increasing excitement of type Ia afferents in muscle spindles [24, 26, 47]. Signals from muscle spindles are directed to motor neurons, which activate the muscles to restore joint position and stimulation can affect the short-latency reflexive mechanism, which is, for example, implemented in maintaining balance during upright standing [48]. On the other hand, proprioceptive feedback from muscle spindles provides information regarding the orientation of the limbs, which is processed in the central nervous system [49, 50], and vibratory stimulation can also influence these long-latency responses [51, 52]. In support of this theory, previous work demonstrated that vibratory stimulation of lower-extremities (e.g., calf muscle and Achilli’s tendon) affects upright balance sway, represented by changes in the body sway behaviors (i.e., larger and faster body sway) [21–23, 25]. Current results showed that vibratory stimulation, probably within the same mechanism, influences dynamic balance during balance recovery by altering proprioceptive performance.

### Effect of stimulation area

Results from the current study suggest that similar to upright standing balance tests, the contribution of ankle and hip muscle proprioceptive information for balance recovery differs across age groups. Considering control young participants with presumably intact proprioceptive sensation, ankle muscles may play the dominant role for balance recovery based on the current findings. Literature supports these findings, showing that among healthy young participants, proprioceptive information from the ankle is crucial for maintaining upright standing balance and dynamic balance recovery [23, 48, 53, 54]. For instance, application of vibratory stimulation on ankle muscles or tendons showed an increase in the body sway during upright standing balance among healthy young adults, which represents a worsened balance because of disturbance of the ankle proprioceptive information [21–23, 25]. Interestingly, previous work also suggest that the same type of ankle proprioceptive disruption showed smaller or no alterations in postural balance among low fall risk older adults [41, 55]. Findings from these studies suggest that ankle proprioceptive information provide the crucial role for upright postural balance in young participants and this dominant role may fade away with aging.

In addition to upright standing balance, research around non-stepping balance recovery assessment demonstrated a dominant ankle involvement (ankle strategy: rotating the body around the ankle joint) for maintaining balance for minor perturbations and dominant hip involvement (hip strategy: flexing the hip and plantar flexing the ankles) for more challenging recoveries [19, 48]. Implementing hip strategy provides the capability for accelerating the center of mass faster than the ankle strategy [56]. Further, comparing young and older age groups, ankle strategy is more frequently employed by young participants, while older adults use hip as the dominant resource for executing the non-stepping recovery [20]. The additional hip involvement (added to ankle involvement) in older adults is to overcome the relatively higher demand for maintaining the upright standing posture [20]. The observed higher involvement of hip joint in maintaining balance suggest a more contribution of hip proprioception as well. Furthermore, older adults may rely more on hip proprioception due to declines in ankle proprioceptive sensitivity, muscle spindle function, and reaction speed, which can affect the ankle’s effectiveness in providing timely balance feedback. This shift is considered compensatory, with hip proprioception providing a more robust balance response under dynamic condition [57]. Previous work, in agreement, reported a positive association between proprioceptive performance of the ankle joint in both young and older adults, while, this association only exists among older adults for the hip joint [14]. The current vibratory stimulation platform provided the opportunity to further investigate the role of ankle and hip proprioception in stepping recoveries. In continuation of previous research, for the first time, we confirmed that hip proprioception may also contribute in stepping recoveries in older adults, even more than ankle proprioception.

### Effects of vibration frequency and treadmill speed

Current findings demonstrated no conclusive difference in balance recovery outcomes between different stimulation frequencies (40 vs. 80 Hz). These frequencies were selected here because previous work on postural upright standing balance showed that in healthy young adults, vibration of ankle muscles within frequencies between 40 and 80 Hz produce the maximal effect on postural balance; however, in frequencies around 40 Hz the vibration effects may not be consistent and vary between healthy participants and high fall risk older adults [38–40]. In our previous work, although not significantly different, we showed that in healthy young participants, 80 Hz stimulation disrupted the balance recovery performance to a larger level compared to 40 Hz stimulation [27]. However, for high fall risk older adults, who may fall between healthy young adults and those with a high risk of falling, no difference was observed between the 40 Hz and 80 Hz stimulations.

Significant differences in balance recovery outcomes were observed for low (0.35 m/s) vs. high (0.7 m/s) treadmill backward perturbation. Among both young and older adult participants, overall, time to full recovery from the perturbation was the outcome that was not significantly different between low and fast speed conditions. This suggest that both healthy young and older adults were able to adjust their recovery behavior and efficiently achieve a steady state walking regardless of the perturbation difficulty. These adjustments were observed as changes in initial response to the perturbation (i.e., decreased reaction time and increased first recovery step length) and an increased trunk involvement (i.e., increased trunk angular rotations). Based on the current findings, on average, the reaction time decreased by 16% and the length of first recovery increased by 26% among all participants when perturbed by faster treadmill speed (Tables 4 and 5). Also, the range of trunk flexion/extension angle during the initial recovery stepping, on average, increased by 56% when exposed to faster treadmill perturbation (Tables 4 and 5). As a result of these adjustment, all participants were able to successfully recover from perturbations; nevertheless, more challenging treadmill perturbation may significantly affect the full recovery process, which should be studied in the future research.

### Limitation and future work

In interpreting the findings of our study, several limitations should be considered, necessitating research to further validate and extend our results. One primary limitation was the lack of inclusion of high risk of fall older adults in our study. Future studies should aim to recruit more diverse cohorts of older adults, particularly those at high risk of falls, to enhance the robustness and applicability of our conclusions regarding the effects of vibratory stimulation on balance recovery. This is especially important, because with aging, the efficacy of muscle spindles declines, due to the natural decline in neural fibers and demyelinization [58–61], leading to a gradual decline in proprioceptive performance [62]. This signal deterioration, to some extent, may be compensated by applying random low-intensity noise (i.e., stochastic resonance), which previously showed promising results for enhancing mechanoreceptor, vision, and vestibular performance [63–65]. Accordingly, the effect of vibratory stimulation on balance recovery, as observed in our previous research for upright standing [41, 55], might be different for high fall risk older adults compared to healthy participants. Further, in this study with the selected treadmill speeds no fall or harness assisted recovery occurred, and therefore, we were unable to investigate vibration effects on balance recovery during more challenging conditions. In the future research, higher treadmill acceleration, as well as slipping behaviors (forward treadmill perturbations) should be studied to complement current findings.

Our study focused on vibratory stimulation of ankle and hip muscles, and the role of sole muscles in dynamic balance performance was not investigated. Future research should explore the effect of sole vibratory stimulation to comprehensively understand its contribution to balance recovery mechanisms, particularly in older adults where sensory deficits may vary across different muscle groups. Lastly, future research should focus on investigating the long-term effects of repetitive and/or long-term vibratory stimulation exposures on proprioceptive responses. Along with advancements in technology, such as wearable devices and virtual reality simulations, vibratory stimulation may offer exciting opportunities to create more ecologically valid assessments of dynamic balance recovery. Integrating these technologies into future studies could provide deeper insights into real-world scenarios and further refine balance training interventions aimed at reducing fall risks among older adults.

## Conclusion and clinical implications

The current study underscores the role of proprioceptive information from ankle and hip muscles in balance recovery across different age groups. We successfully perturbed both young and older adult participants and assessed balance recovery kinematics. Among young controls, both 40 and 80 Hz vibration of ankle muscles significantly increased the reaction and, consequently, increase the length of initial reactive step. Among older adult, application of vibratory stimulation on hip muscles significantly increased the reaction time and the length of initial reactive step. We confirmed that the location of vibratory stimulation plays a significant role on its effects on balance recovery performance; stimulation on ankle significantly influence balance recovery among healthy young control, while it showed no effect when placed on hip muscles. On the other hand, while vibratory stimulation on ankle showed no effect on balance recovery among older adults, it significantly influenced balance recovery when hip muscles were exposed to it. This age-related disparity underscores the importance of tailored interventions to enhance proprioceptive and motor responses in older adults, potentially mitigating their heightened fall risk and improving overall balance.
